# Distinct Functional Interactions between Actin Isoforms and Nonsarcomeric Myosins

**DOI:** 10.1371/journal.pone.0070636

**Published:** 2013-07-26

**Authors:** Mirco Müller, Ralph P. Diensthuber, Igor Chizhov, Peter Claus, Sarah M. Heissler, Matthias Preller, Manuel H. Taft, Dietmar J. Manstein

**Affiliations:** 1 Institute for Biophysical Chemistry, Hannover Medical School, Hannover, Germany; 2 Institute of Neuroanatomy, Hannover Medical School, Hannover, Germany; University of Heidelberg Medical School, Germany

## Abstract

Despite their near sequence identity, actin isoforms cannot completely replace each other *in vivo* and show marked differences in their tissue-specific and subcellular localization. Little is known about isoform-specific differences in their interactions with myosin motors and other actin-binding proteins. Mammalian cytoplasmic β- and γ-actin interact with nonsarcomeric conventional myosins such as the members of the nonmuscle myosin-2 family and myosin-7A. These interactions support a wide range of cellular processes including cytokinesis, maintenance of cell polarity, cell adhesion, migration, and mechano-electrical transduction. To elucidate differences in the ability of isoactins to bind and stimulate the enzymatic activity of individual myosin isoforms, we characterized the interactions of human skeletal muscle α-actin, cytoplasmic β-actin, and cytoplasmic γ-actin with human myosin-7A and nonmuscle myosins-2A, -2B and -2C1. In the case of nonmuscle myosins-2A and -2B, the interaction with either cytoplasmic actin isoform results in 4-fold greater stimulation of myosin ATPase activity than was observed in the presence of α-skeletal muscle actin. Nonmuscle myosin-2C1 is most potently activated by β-actin and myosin-7A by γ-actin. Our results indicate that β- and γ-actin isoforms contribute to the modulation of nonmuscle myosin-2 and myosin-7A activity and thereby to the spatial and temporal regulation of cytoskeletal dynamics. FRET-based analyses show efficient copolymerization abilities for the actin isoforms *in vitro*. Experiments with hybrid actin filaments show that the extent of actomyosin coupling efficiency can be regulated by the isoform composition of actin filaments.

## Introduction

Mammalian actin isoforms are highly conserved and ubiquitously found in eukaryotic cells. The 42 kDa globular actin monomer is composed of four subdomains and can assemble into thin filaments or microfilaments (F-actin). Six actin isoforms can be distinguished: three α-actin isoforms (α-skeletal muscle, α-cardiac muscle and α-vascular), one β-isoform (β-cytoplasmic) and two γ-isoforms (γ-cytoplasmic and γ-smooth muscle). Only subtle sequence variations distinguish the isoactins. The amino acid sequence of α-actin differs from cytoplasmic actin isoforms in more than 20 residues that are spread over the entire molecule. In contrast, differences between β- and γ-actin are restricted to the N-terminus – Asp^2^-Asp^3^-Asp^4^-Ile^5^ (β-actin) and Glu^2^-Glu^3^-Glu^4^-Ile^5^ (γ-actin). The N-terminal sequences of α-skeletal and α-cardiac actin correspond to Asp^3^-Glu^4^-Asp^5^-Glu^6^ and Asp^3^-Asp^4^-Glu^5^-Glu^6^ [1]. Actin isoforms are essential for a wide range of physiological functions. The four muscle actins are restricted to tissues with high tonic activity such as striated heart muscle, skeletal muscle or smooth muscle of blood vessels, gut wall and the urogenital system [2]. By contrast, cytoplasmic actins are ubiquitous and play a pivotal role in cell motility, intracellular transport, cell shape maintenance or mitosis [3]. They undergo spatial and temporal segregation during the formation of stress fibers and actin-based cell protrusions [4], [5]. The resulting structures are regulated by specific actin-binding proteins [6], [7]. There is strong evidence that the isoactins cannot substitute for each other [8], [9], [10], [11], indicating functional intracellular specialization [12], [13]. The β-isoform preferably localizes in stress fibres, circular bundles and at cell-cell contacts as an unbranched filamentous array. Dependent on cellular activities, γ-actin displays a more variable distribution. It is mainly organized as a branched meshwork with cortical and lamellar localization in moving cells, but can colocalize with β-actin in lamellipodia or be recruited into stress fibres [3], [12]. Knockout studies in mice revealed, that both cytoplasmic actin isoforms are required for stereocilia maintenance in hair cells [14]. Aberrant expression of particular isoactins is a significant feature of adaptive and pathological alterations in e.g. wound healing, cardiovasular diseases, myopathies or tumor metastasis [2], [15]. Mutations of β-actin cause pleiotropic diseases and can be linked to neutrophil dysfunction (mutation E364K) [16], malformations, deafness, and delayed-onset dystonia (R183W) [17]. In addition, β-actin mutations are associated with metastasis [18] and tumours like the diffuse large B-cell lymphoma [19], [20]. Multiple missense mutations of γ-actin have been described (T89I, K118M, K118N, I122V, E241K, P264L, T278I, P332A, V370A), all of them are associated with autosomal dominant non-syndromic sensorineural progressive hearing loss [21], [22], [23], [24], [25], [26], [27].

Myosin isoforms are members of a structurally and functionally diverse superfamily of mechanoenzymes. They use actin-activated ATP turnover to trigger cyclic conformational changes leading to force generation and directional movement. Members of the nonmuscle myosin-2 (NM-2) family act as central regulators of the highly dynamic eukaryotic cytoskeleton. In vertebrates, three isoforms can be distinguished: nonmuscle myosin-2a (NM-2A), -2B (NM-2B), and -2C (NM-2C). The complexity of the NM-2 proteome is enhanced by alternative splicing of the NM-2B and -2C isoforms. The enzymatic properties, subcellular and tissue-specific localizations of NM-2 isoforms and their splice variants are distinctive [28], [29], [30] but they can substitute for each other to a certain extent [31]. NM-2 isoforms are required for cell migration, cell adhesion, vesicle transport, and cytokinesis (reviewed by Heissler and Manstein [32]). NM-2A mutations are associated with congenital macrothrombocytopenia, deafness, progressive nephropathy, and presenile cataracts [33]. In addition, alterations of NM-2A activity or expression level contribute to tumor invasion and [34], [35], [36] metastasis. NM-2B is only indirectly linked to pathologies including neurodegenerative diseases [32]. Mutations in NM-2C are associated with deafness, distal myopathy and peripheral neuropathy [37], [38]. Myosin-7A belongs to the unconventional human myosins. It is involved in mechano-electrical transduction and stereocilia morphogenesis in cochlear hair cells [39], [40] and has been associated with lysosomal as well as neuroretinal melanosome, phagosome and opsin trafficking [41], [42], [43], [44]. Mutations of myosin-7A are causative for non-syndromic hearing loss and Usher syndrome type 1B [45], [46].

The ability of NM-2 and myosin-7A motors to support multiple functions within the cytosol is closely linked to the occurrence of the cytoplasmic β- and γ-actin. Their common physiological tasks and relation to similar pathologies suggests a special mode of interaction between cytoplasmic actin and myosin isoforms. Here, we describe the modulating effect α-, β- and γ-actin have on the functional properties of cytoplasmic myosins.

## Materials and Methods

### Reagents

Restriction enzymes, DNA-Polymerase, DNA-modifying enzymes (Fermentas), NHS-Rhodamine labeling reagents (5-(and 6)-carboxytetramethylrhodamine, *N*-Hydroxysuccinimide ester; Pierce Biotechnology), goat anti-mouse horseradish-peroxidase and SuperSignal West Femto Maximum Sensitivity Substrate were purchased from Thermo Fisher Scientific. FRET (Förster resonance energy transfer) labeling dyes and NPE-caged ATP (Adenosine 5′-Triphosphate, P3-(1-(2-Nitrophenyl)Ethyl) Ester) were from Invitrogen (Life Technologies). Penta-His antibody and Ni^2+^-NTA were purchased from QIAGEN. Standard reagents were from Sigma-Aldrich.

### Plasmid construction

The human gelsolin C-terminal half G4-6 was subcloned from shuttle vector pKN172 into the cold-shock expression vector pCOLD II (Takara Bio, Inc.) using the restriction sites for *Bam*HI and *Hind*III after introduction of a polyhistidine tag using the primers 5′-GATCCCATCACCATCATCACCATC ACG -3′ and 5′-GATCCGTGATGGTGATGATGGTGATGG-3′. The baculovirus transfer vectors for β- and γ-actin were produced as follows. The cDNAs for human cytoplasmic actins were amplified by PCR using the primers: 5′-GCCTCGAGATGGATGATGATATCGCCGCG-3′ (N-terminal β-actin), 5′-GCCTCGAGATGGA AGAAGAGATCGCCGCGCTGGTCATTGACAATGGC-3′ (N-terminal γ-actin), and 5′- GCATGCATCTAG AAGCATTTGCGGTGGACG-3′(C-terminal, β- and γ-actin). The DNA fragments were cloned into pFastBac Dual vectors (Invitrogen) by usage of the restriction enzymes *Xho*I and *Nsi*I under the p10 promoter for expression in the baculovirus/*Sf*9 system. The baculovirus transfer vectors encoding the motor domains of human nonmuscle myosin-2A (residues 1–775), -2B (residues 1–782), and -2C1 splice variant (residues 1–807), each fused with two *Dictyostelium discoideum* α-actinin repeats acting as an artificial lever arm and an octahistidin-tag, were generated as described previously [47]. The myosin-7a motor domain construct (amino acids 1–747) was cloned similarly but fused with an EYFP fluorescence marker additionally to the artificial lever arm [48]. The transfer vectors encoding cytoplasmic actins and NM-2 isoforms were transformed in DH10Bac *E. coli* cells to generate recombinant bacmids. After isolation and confirmation by PCR, the respective bacmids were transfected into *Sf*9 (*Spodoptera frugiperda*) insect cells using Cellfectin II (Invitrogen). Recombinant virus was amplified according to the manufacturer's protocol, *Sf*9-cells were infected and harvested 3 days *post infectionem* and stored at −80°C. All plasmids were verified by sequencing. DNA sequences were analyzed in DNASTAR Lasergene Core Suite software (DNAstar, Inc.).

### Protein preparation

The C-terminal gelsolin half G4-6 was produced as described by Ohki et al. [49]. Typically, the purification of G4-6 yielded 50–100 mg pure protein per liter *E. coli* culture. Purification of mouse tropomyosin α-1 chain (Tm) was performed according to Coulton et al. [50] and typically yielded 60 mg per liter culture. Tag-free cytoplasmic human β- and γ-actin were purified as G-actin (globular or monomeric actin) by affinity chromatography using the G4-6 gelsolin deletion mutant according to Ohki et al. [49]. Mammalian skeletal muscle α-actin was purified from rabbit as described previously [47], [51]. For selected experiments, monomeric α-actin was further purified by G4-6 affinity chromatography as described above. Actins were polymerized by the addition of 2 mM MgCl_2_ and 0.1 M KCl for 3 h at 21°C and then used in the assay. Actin which was not used immediately was kept on ice until usage for no longer than 3 days. Sufficient polymerization ability of the actin isoforms was checked by sedimentation of F-actin at 100.000 *g* and subsequent SDS-PAGE (sodium dodecyl sulfate polyacrylamide gel electrophoresis) of the supernatant and the pellet, which was resolved in an equal volume. Recombinant human myosin-2A, -2B and 2C1 and myosin-7A constructs were purified as described previously [30], [48]. Proteins were supplemented with 3% sucrose (actin, Tm) or 10% trehalose (myosin), flash frozen in liquid N_2_ and stored at −80°C.

### Gel electrophoresis, Immunoblots and IEF

Polyacrylamide gel electrophoresis (10%) in the presence of SDS was used to show the homogeneity of the actin preparations. For immunoblotting, protein samples were separated by SDS-PAGE, transferred onto nitrocellulose membrane and blocked with nonfat dry milk (5% w/v) in TBST (20 mM Tris-HCl pH 7.5, 150 mM NaCl, 0.05% (v/v) Tween-20). The membrane was incubated overnight with monoclonal anti-muscle-actin, anti-β-actin, or anti-γ-actin mouse antibody in TBST (1∶1000 dilutions). Monoclonal muscle specific actin antibody Ab-4 (mouse, clone HHF35) was from Thermo Fisher Scientific, monoclonal anti-β-actin (mouse, clone AC-74) and anti-γ-actin (mouse, clone 2-2.1.14.17) antibody were purchased from Sigma-Aldrich. After washing with TBST it was kept for 1 h at room temperature with horseradish peroxidase conjugated secondary antibody. Detection was performed by means of chemiluminescence. Isoelectric focusing (IEF) electrophoresis (pH range 4–7) was performed using the ZOOM IPGRunner Kit (Life Technologies) according to the manufacturer's protocol.

### LC-MS analysis

Sample preparation and processing (liquid chromatography, LC) for mass spectrometry (MS) analysis was performed as described previously [52] with the exception that proteins were alkylated by addition of 2% acrylamide. Peptide samples were separated with a nano-flow ultra-high pressure liquid chromatography system (RSLC, Thermo Scientific). The RSLC system was coupled online via a Nano Spray Flex Ion Source II to an LTQ-Orbitrap Velos mass spectrometer (both from Thermo Scientific). Raw data were processed using Proteome Discoverer software (version 1.2, Thermo Scientific), the Mascot search engine and human entries of the SwissProt/Uniprot database. Acetylation, oxidation, deamidation, arginylation and propionylation were used as possible modifications. Peptides with a peptide ion score above 30 and a false discovery rate below 0.05 were considered.

### FRET experiments

Fluorescence labeling of α-, β-, and γ-actin and FRET copolymerization assay were performed as follows. Each isoform was independently labeled with 1,5-IAEDANS (5-((((2-Iodoacetyl)amino)ethyl)amino) Naphthalene-1-Sulfonic Acid, λ_Emission,max_ = 490 nm) and 5-IAF (5-Iodoacetamidofluorescein, λ_Emission,max_ = 520 nm). Briefly, 1 mg/ml monomeric actin in G-actin buffer (10 mM Tris-HCl pH 8.0, 0.2 mM CaCl_2_, 1 mM DTT, 0.5 mM ATP) was mixed with 5-fold molar excess of labeling dye and incubated at room temperature for 4 hours. After polymerization (see also *protein preparation*), F-actin was pelleted by centrifugation at 100.000 *g* for 3 hours. The resulting pellets were homogenized in G-actin buffer and incubated at 4°C over night. To remove actin unable to depolymerize the solution was centrifuged at 100.000 *g* for 1 hour. The G-actin concentration in the supernatant and the extent of actin labeling were determined according to the manufacturer's protocol. Finally, an equimolar mixture of 1,5-IAEDANS and 5-IAF labeled actin was polymerized and fluorescence emission spectra were measured at a concentration of 1.3 µM in an Varian Cary Eclipse spectrofluorometer (λ_Excitation,IAEDANS_ = 336 nm).

### ATPase measurements

Comparative steady-state kinetics of the various myosin isoforms stimulated by α-, β-, and γ-actin homopolymers or copolymers were measured with the NADH-coupled assay as described previously [53]. Using an ATP regeneration system consisting of 0.05 mg/ml pyruvate kinase, 0.5 mM phosphoenolpyruvate, 0.02 mg/ml lactate dehydrogenase measurements were performed at 25°C in a buffer containing 25 mM HEPES (pH 7.4), 5 mM MgCl_2_, 0.2 mM NADH, 1 mM ATP and 0.5 mM DTT. Myosin concentrations were varied between 0.2 and 1.0 µM and F-actin from 0–100 µM. Following the NADH oxidation by measuring the decrease in absorption at 340 nm (ε = 6220 M^−1^ cm^−1^), the ATPase rates were determined by linear curve fitting and the basal ATPase activity of myosin was subtracted from the actin-activated data.

### Flash photolysis experiments

Transient kinetic measurements were performed at 30°C using a flash photolysis system as described previously [54]. 1 µM myosin was mixed with 5 µM F-actin and 0.5 mM caged-ATP (cATP) in a buffer containing 50 mM KP_i_ pH 7.4, 400 mM KCl, 5 mM MgCl_2_ and 10 mM DTT. The third harmonics (355 nm) of a Surelite II-10 NdYAG laser was used for photolysis of cATP. The amount and time of ATP release was monitored from the increase of absorbance at 405 nm due to the formation of the excited aci-nitro intermediate. The minimal concentration of free ATP was at least 5-fold higher than the myosin concentration. This allows analysis of the reaction within pseudo-first order approximation. ATP induced dissociation of myosin from F-actin and following reassociation after full hydrolysis of ATP was monitored as the change of light scattering signal (λ>400 nm).

### In vitro motility assay

F-Actin isoforms were stabilized and visualized by decoration with fluorescently labeled Tm-dimers because the behavior of native actin filaments in their interaction with NM-2 isoforms can be altered when stabilized with phalloidin [47]. The labeling was performed using the NHS-Rhodamine Labeling Kit (Pierce) according to the manufacturer's protocol. Using assay buffer AB (25 mM Imidazole pH 7.4, 25 mM KCl, 4 mM MgCl_2_, 1 mM EGTA), 10 µM F-actin (α-, β-, and γ-actin, respectively) was incubated with an equimolar amount of labeled Tm for at least 1 h at 21°C or overnight on ice. The actin-Tm complex was then sedimented at 100.000 *g* for 30 min, washed with AB-buffer and gently resuspended with the same buffer by thorough pipetting. The complex was diluted to 50 nM F-actin directly before usage. Standard *in vitro* motility assays were performed at 30°C using an Olympus IX70 inverted fluorescence microscope according to published protocols [30], [55]. Myosin concentration in the assay was 1 mg/ml. Inactive myosin heads were removed by actin affinity purification. We increased the incubation time of Tm-actin with myosin to 10 min and included 0.5% methylcellulose to prevent diffusion of Tm away from actin and Tm-actin from the myosin substratum [56]. The movement of at least 150 actin filaments per observation area was recorded over a length of 50 frames with an exposure time of 1000 ms and a cycle time of 2000 ms using CellR Software version 2.8 (Olympus). Automated actin filament tracking was performed with the program DiaTrack 3.02 (Semasopht/INDEC Biosystems) and the average sliding velocity was determined by analysis of the Gaussian distribution with Origin 8.5 (OriginLab).

### Computational and statistical analysis

Data analysis and graph plotting were performed using Origin 8.5 (OriginLab) and GraphPad Prism version 5.02 (GraphPad Software). Unpaired *t*-test was employed for statistical analysis of the *in vitro* motility data using GraphPad Prism. If not otherwise specified, each single measure was repeated at least three times and errors were calculated as standard errors.

## Results

**Figure 1:**
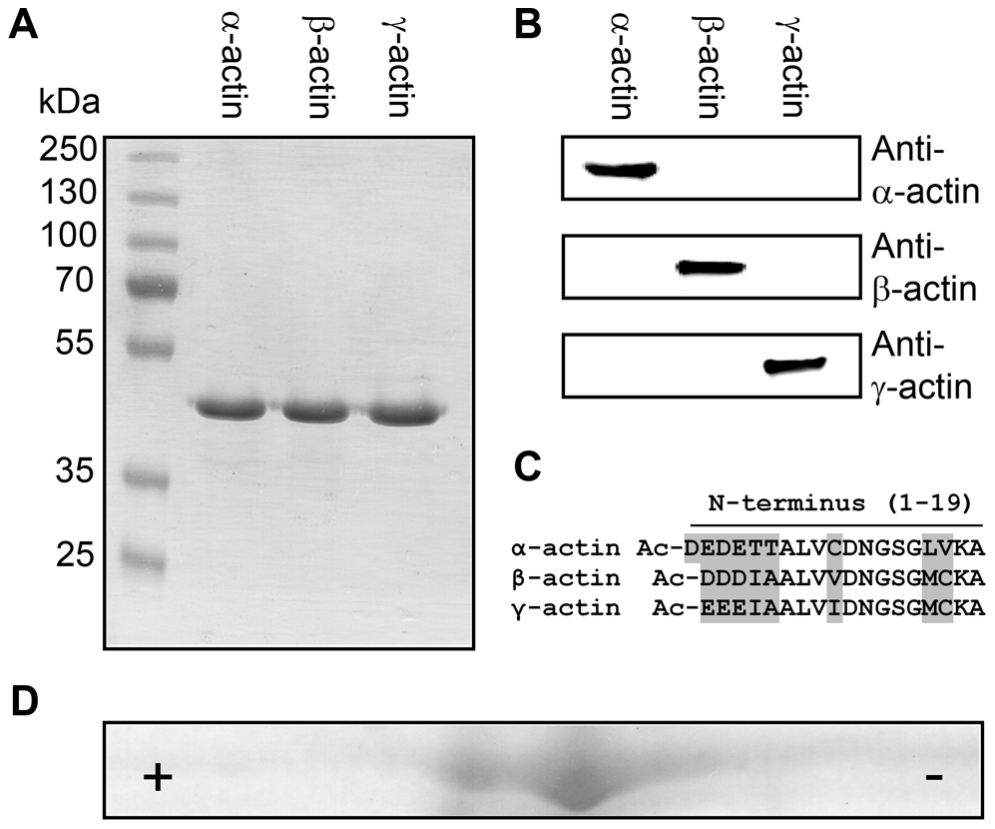
Purification of actin isoforms. (A) SDS-PAGE of purified actin isoforms. (B) Immunoblot-based identification of actin isoforms. The actin antibodies used show no cross-reactivity. (C) Actin isoforms display subtle differences primarily located at their N-terminus as shown in the protein sequence alignment. Sequence variations at the N-terminus of α-, β- and γ-actin are marked in grey. Mature actin isoforms are acetylated (Ac) at their N-terminus (D) 2D-gelelectrophoresis of purified γ-actin (IEP 5.31) containing ∼10% co-purified insect actin (IEP 5.29). The acidic (+) and the basic end (−) are marked.

The cytoplasmic actin isoforms were produced in *Sf*9 cells and purified to homogeneity (Figure 1A). Immunoblots were used to verify the purity of the particular isoactins (Figure 1B). Although actin isoforms display only subtle N-terminal differences (Figure 1C), the monoclonal antibodies used are highly specific for this region and show no cross-reactivity with other isoactins. IEF was performed to determine the amount of contaminating insect actin. Due to differences in their isoelectric points (IEP), the more basic γ-actin (IEP 5.31) can be separated from the more acidic insect actin (IEP 5.29) as depicted in Figure 1D. Insect and human β-actin have similar IEPs and separate less well. IEF and MS analyses indicate that the level of contamination of human cytoplasmic actins with endogenous actin from insect cells is in the range of 5–15% (N = 3).

**Figure 2:**
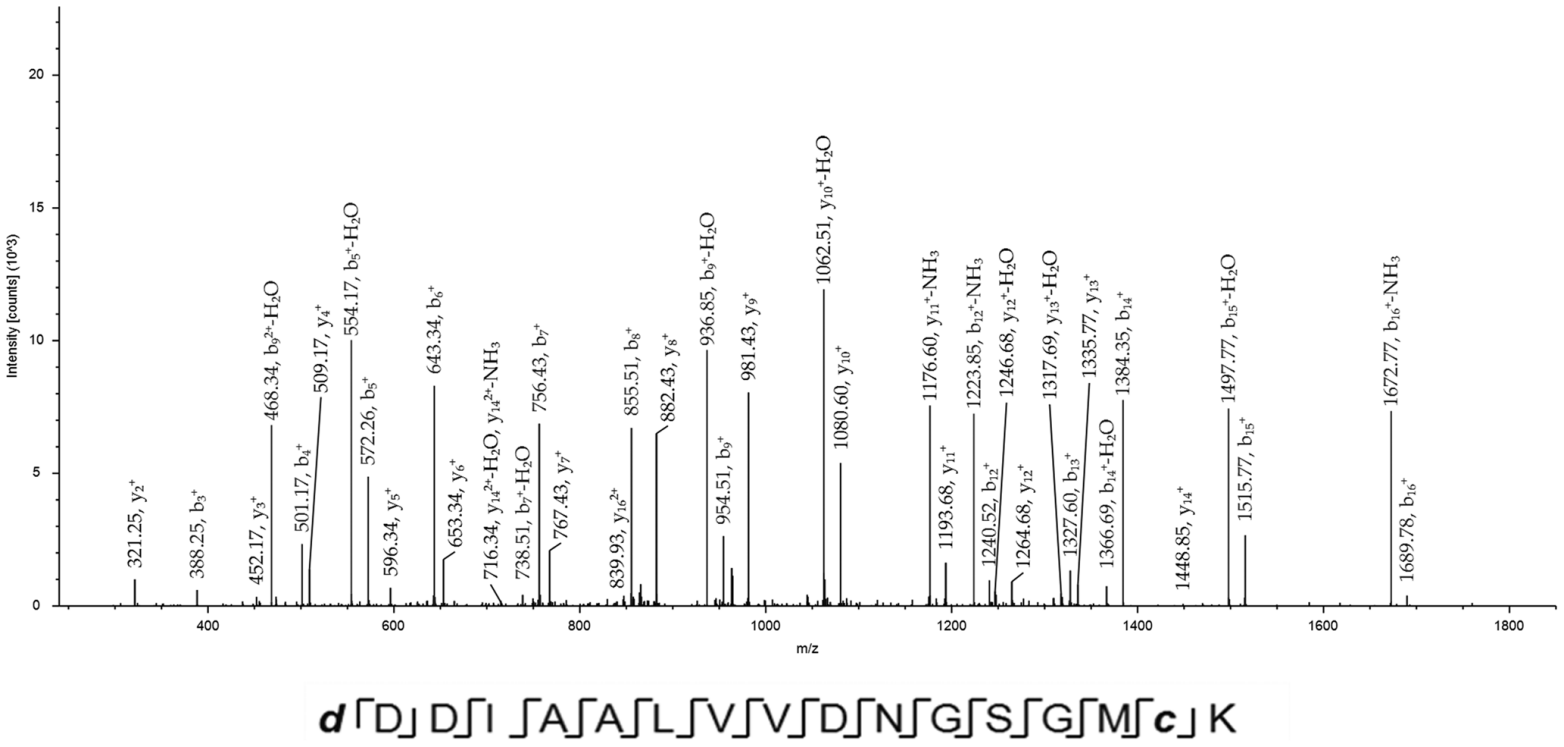
β- and γ-actin are N-terminally acetylated. Mass spectrum of the N-terminal peptide of β-actin. The protonated molecular ion (MH^+^) of the identified peptide has a mass of 1835.8 Da and is modified by removal of Met1 (m = 1722.9 Da), acetylation of Asp1 (indicated as d, Δm = +42 Da) and due to sample processing by an artificial propionamidation of cysteine (indicated as c, Δm = +71 Da). Similar results were obtained with γ-actin (spectrum not shown).

The purified recombinant proteins are identical in sequence to the native actin isoforms. MS analyses showed that both recombinant actin isoforms purified from *Sf*9 cells are post-translationally modified at their N-terminus. We identified trypsin digested β-actin with a sequence coverage of >99% (56 peptides, score 56,000) and γ-actin with a coverage of >93% (52 peptides, score 59,000). The predominant species (>95%) has undergone N-terminal removal of Met^1^ and acetylation at Asp^2^ (β-actin) or Glu^2^ (γ-actin). Additionally, we detected N-terminal peptides with residual amounts (<5%) of acetylated Met^1^, a modification required for removal of the first residue. A mass spectrum of the modified N-terminal peptide from β-actin is shown in Figure 2.

### ATP induced dissociation and turnover kinetics of specific actomyosin complexes

**Figure 3:**
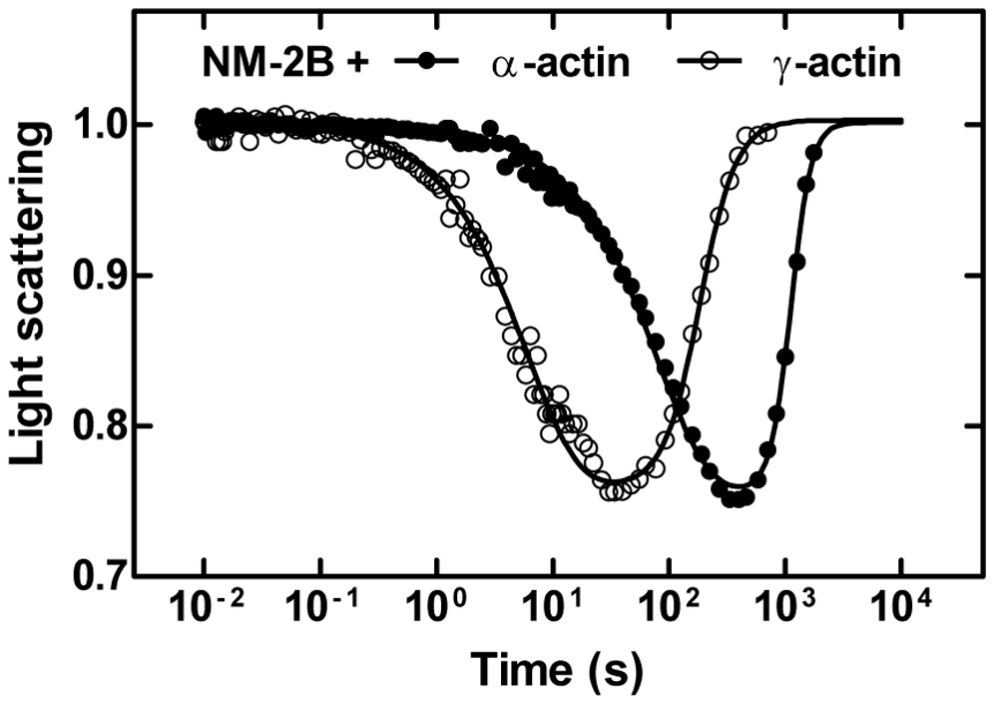
Time-dependent dissociation and association of NM-2B from filamentous α- and γ-actin. The light scattering signal normalized to the initial value shows the time-dependent dissociation and association of the actomyosin complex formed by NM-2B and α- or γ-actin. The initial drop in the light scattering signal follows the release of ATP from caged-ATP by a flash of UV-laser. The single exponential reduction in scattering intensity monitors the ATP-induced dissociation of myosin from F-actin. The following restoration of the light scattering signal is caused by rebinding of myosin to the actin filament after ATP hydrolysis is completed. As shown for NM-2B, γ-actin causes faster dissociation and association compared to α-actin. Note the logarithmic time scale on the graph.

**Figure 4:**
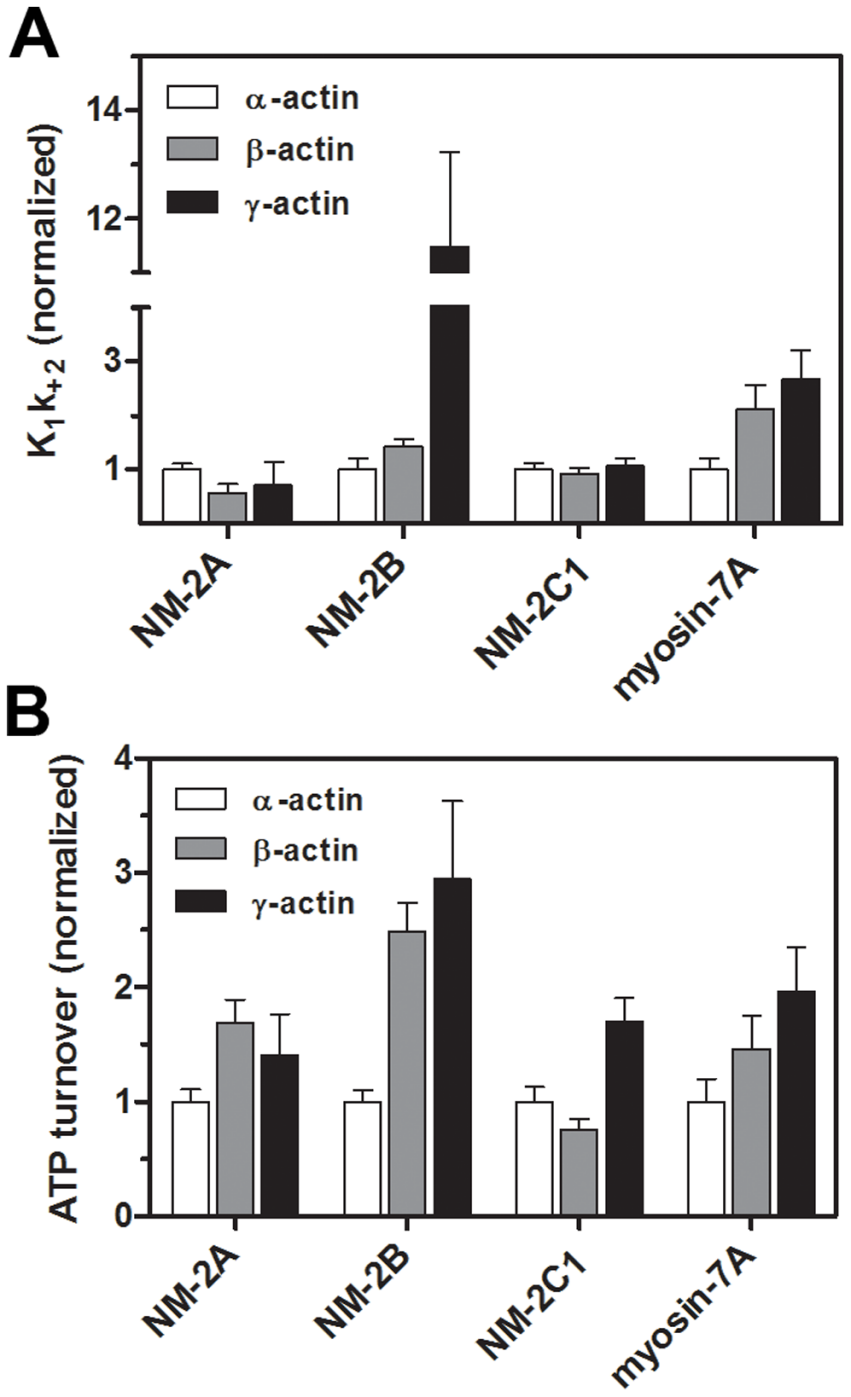
Flash photolysis experiments with nonmuscle myosin and actin isoforms. (A) The graphs show the relative changes of the apparent second order rate constants K_1_k_+2_. P-values of unpaired *t*-tests were <0.05 for the combinations NM-2A (α- and β-actin), NM-2B (α- and β-/γ-actin), and myosin-7A (α- and β-/γ-actin). (B) ATP turnover rates of nonmuscle myosin isoforms interacting with β- or γ-actin. P-values of unpaired *t*-tests were <0.05 for the combinations NM-2A (α- and β-/γ-actin), NM-2B (α- and β-/γ-actin), NM-2C1 (γ- and α-/β-actin), and myosin-7A (all combinations). Rates were normalized using α-actin as a reference. The ATP-induced dissociation is accelerated for the complexes NM-2B/β-/γ-actin and myosin-7A/β-/γ-actin. The interaction of cytoplasmic actins and NM-2 or myosin-7A isoforms leads to 2- to 3-fold increased catalytic activities. Reference values for K_1_k_+2_ (µM^−1^ s^−1^) as determined with α-actin are as follows: NM-2A, 0.14±0.003; NM-2B, 0.24±0.02; NM-2C1, 0.89±0.01; myosin-7A, 0.44±0.01 (see also [32], [48]).

We used flash photolysis of an inert ATP precursor (caged-ATP) to initiate the dissociation of NM-2/F-actin and myosin-7A/F-actin complexes and subsequent reaction stages (Figure 3). The fast dissociation of the actomyosin complex upon ATP binding can be monitored by light scattering, which is highly sensitive to changes in size and molar mass of protein complexes. The apparent dissociation rate constant k_obs_ was obtained by fitting the data to a single exponential function. The ATP concentration released by each flash and the associated values for k_obs_ were determined and used to calculate the apparent second order rate constant **K_1_k_+2_** for nucleotide induced dissociation of the actomyosin complex. **K_1_** is the fast diffusion limited equilibrium constant for ternary complex formation. ATP binding is followed by an irreversible conformational transition of myosin (**k_+2_**). Once the hydrolysis of the released ATP has been completed, the actomyosin complex reforms at the end of an extended steady-state phase. The transition from steady-state was approximated by a sigmoidal curve and the midpoint was used for calculation of the ATP turnover rate. The resulting values for **K_1_k_+2_** and ATP turnover, as measured with 5 µM F-actin, are summarized in Figure 4A and 4B. Compared to the situation observed in the presence of α-actin, NM-2B and myosin-7A show 11-fold and ∼2-fold increases in **K_1_k_+2_** in the presence γ-actin. In the case of myosin-7A, a ∼2-fold increase was also observed in the presence of β-actin. The rate of ATP-induced dissociation of complexes comprising NM-2A and -2C1 is not increased if α-actin is replaced by β- or γ-actin. With regard to ATP hydrolysis rates, almost all myosin isoforms tested display a significant increase in the presence of the cytoplasmic isoactins. The largest change was observed for the combination NM-2B/γ-actin, showing a 3-fold increase in ATP turnover (Figure 4B). Only the combination of NM-2C1 with β-actin showed no increase in ATP turnover.

### Cytoplasmic actins enhance the coupling efficiencies of NM-2 and myosin-7A

**Figure 5:**
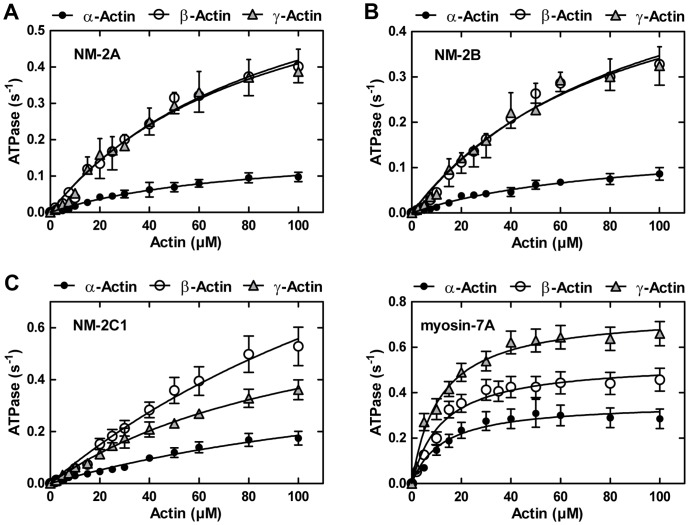
Cytoplasmic actins enhance nonmuscle myosin-2 and myosin-7A efficiency. Steady-state ATPase activities of (A) NM-2A, (B) NM-2B, (C) NM-2C1 and (D) myosin-7A measured as a function of actin concentration (α-, β-, and γ-F-actin). The ATPase activity in the absence of F-actin was subtracted from the actin-activated data. Values for **K_actin_** and **k_cat_** were calculated from a hyperbolic fit (ATPase activity = (**k_cat_** [F-actin])/(**K_actin_**+[F-actin])) to the data (see Table 1).

Steady-state ATPase measurements of NM-2 and myosin-7A in the presence of 1–100 µM filamentous α-, β- and γ-actin were performed to determine the maximal ATP turnover rate (**k_cat_**), the actin concentration required for half-maximal activation (**K_actin_**), and the apparent second order rate constant for F-actin binding in the presence of ATP (**k_cat_/K_actin_**). Based on the values determined for **k_cat_**, β-actin is 3 to 4-fold more potent in the activation of ATP turnover by NM-2 isoforms than α-actin (Figure 5, Table 1). The level of activation induced by γ-actin is 1.5-fold (NM-2C1) and 4-fold (NM-2A and -2B) greater compared to α-actin. The choice of isoactins interacting with NM-2 isoforms appears to have only a minor effect on **K_actin_**. However, due to the high **K_actin_**-values displayed by the NM-2 isoforms and technical limitations of the assay, the respective values should be treated only as rough estimates [30]. For each actomyosin combination, we determined the second order rate constant **k_cat_/K_actin_** (µM^−1^ s^−1^), which is a direct measure of the coupling efficiency between the actin– and nucleotide–binding sites of myosin. Values for **k_cat_/K_actin_** can be determined accurately at actin concentrations much smaller than **K_actin_** from the initial slope of the Michaelis-Menten-plot (Figure 5 and Table 1). The coupling efficiencies of NM-2 isoforms with β- or γ-actin are significantly greater (2-fold to 4-fold) compared to α-actin. The stimulation of myosin-7A ATPase and its coupling efficiency (Figure 5, Table 1) is highest with γ-actin (2-to 3-fold over α-actin).

**Table 1:** Summary of steady-state kinetic parameters of NM-2A, -2B, -2C1 and myosin-7A activated by actin isoforms.

		α-actin	β-actin	γ-actin
NM-2A	K_app,actin_ (µM)	80.2±11.5	94.6±16.6	88.0±16.2
	k_cat_ (s^−1^)	0.18±0.01	0.81±0.08	0.77±0.08
	Coupling efficiency*	0.002±0.0005	0.008±0.001	0.008±0.001
NM-2B	K_app,actin_ (µM)	82.0±13.9	95.1±20.2	86.9±16.8
	k_cat_ (s^−1^)	0.15±0.01	0.67±0.08	0.63±0.07
	Coupling efficiency*	0.002±0.001	0.007±0.001	0.007±0.001
NM-2C1	K_app,actin_ (µM)	>100	>100	>100
	k_cat_ ** (s^−1^)	0.17±0.02	0.53±0.07	0.36±0.04
	Coupling efficiency*	0.003±0.0005	0.007±0.001	0.006±0.001
Myosin-7A	K_app,actin_ (µM)	12.3±2.3	12.3±1.8	11.3±1.2
	k_cat_ (s^−1^)	0.35±0.01	0.53±0.02	0.75±0.02
	Coupling efficiency*	0.02±0.001	0.04±0.0015	0.06±0.001

* The second order rate constant k_cat_/K_app,actin_ (µM^−1^ s^−1^) indicates the coupling efficiency and was obtained from the initial slope of the steady-state ATPase activity *versus* actin concentration plot.

** Activities of NM-2C1 isoforms in the presence of 100 µM F-actin. With regard to k_cat_, p-values of unpaired *t*-tests were ≤0.01 for the combinations α- and β-/γ-actin for all NM-2 isoforms and myosin-7A.

The k_cat_-values for β- and γ-actin were different for NM-2C1 (p = 0.025) and myosin-7A (p = 0.005). No significant differences were found for K_app,actin_ (p≥0.25 for all combinations). Coupling efficiencies differ significantly (p≤0.05) between α-actin and cytoplasmic actins for all myosin isoforms shown in the table. The coupling efficiencies of β- and γ-actin were different for myosin-7A (p<0.001).

### NM-2 isoforms slide cytoplasmic β- and γ-actin at higher velocities than α-skeletal actin

Fluorescently labeled Tm was used to visualize and stabilize F-actin. The results of *in vitro* motility experiments that were performed with Tm decorated F-actin show that β- and γ-actin support significantly higher motile activity (10–25%) of NM-2A, -2B and -2C1, compared to the velocities observed in the presence of α-actin (Table 2). The sliding velocity of each actomyosin combination was repeatedly determined in fresh flow cells and the mean ± S.E.M. was calculated. P-values of unpaired *t*-tests were <0.05 for the combinations NM-2A/β-actin and NM-2A/γ-actin and <0.01 for all other combination of NM-2 with β- or γ-actin when compared to the respective NM-2/α-actin complexes.

**Table 2:** Motility rates of NM-2 and actin isoforms.

Motility rates (nm s^−1^)	α-actin	β-actin	γ-actin
NM-2A	76.3±3.0 (N = 11)	84.2±1.2 (N = 11)	94.6±2.9 (N = 8)
NM-2B	30.1±0.5 (N = 9)	35.2±0.8 (N = 10)	32.7±1.0 (N = 8)
NM-2C1	51.7±2.1 (N = 13)	60.9±1.1 (N = 21)	61.2±1.1 (N = 13)

Motility rates of myosin-7A with actin isoforms were not determined. N: number of experiments, errors indicate S.E.M.

### Formation and functional behavior of isoactin copolymers

**Figure 6:**
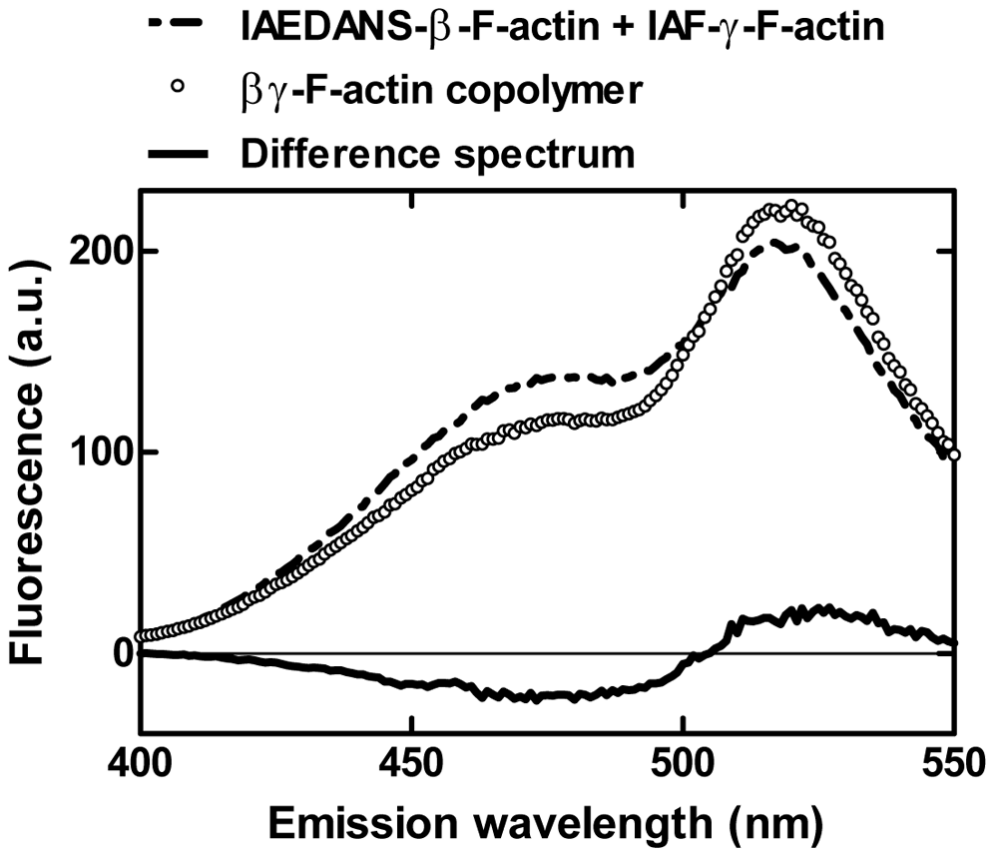
FRET copolymerization analysis of actin isoforms. Fluorescence emission spectra of IAEDANS-β-actin and IAF-γ-actin, both separately polymerized and after copolymerization. The donor fluorescence (IAEDANS) peaks around 490 nm and decreases significantly upon copolymerization whereas the fluorescence of the acceptor (IAF) at 520 nm apparently increases as shown in the difference spectrum.

**Figure 7:**
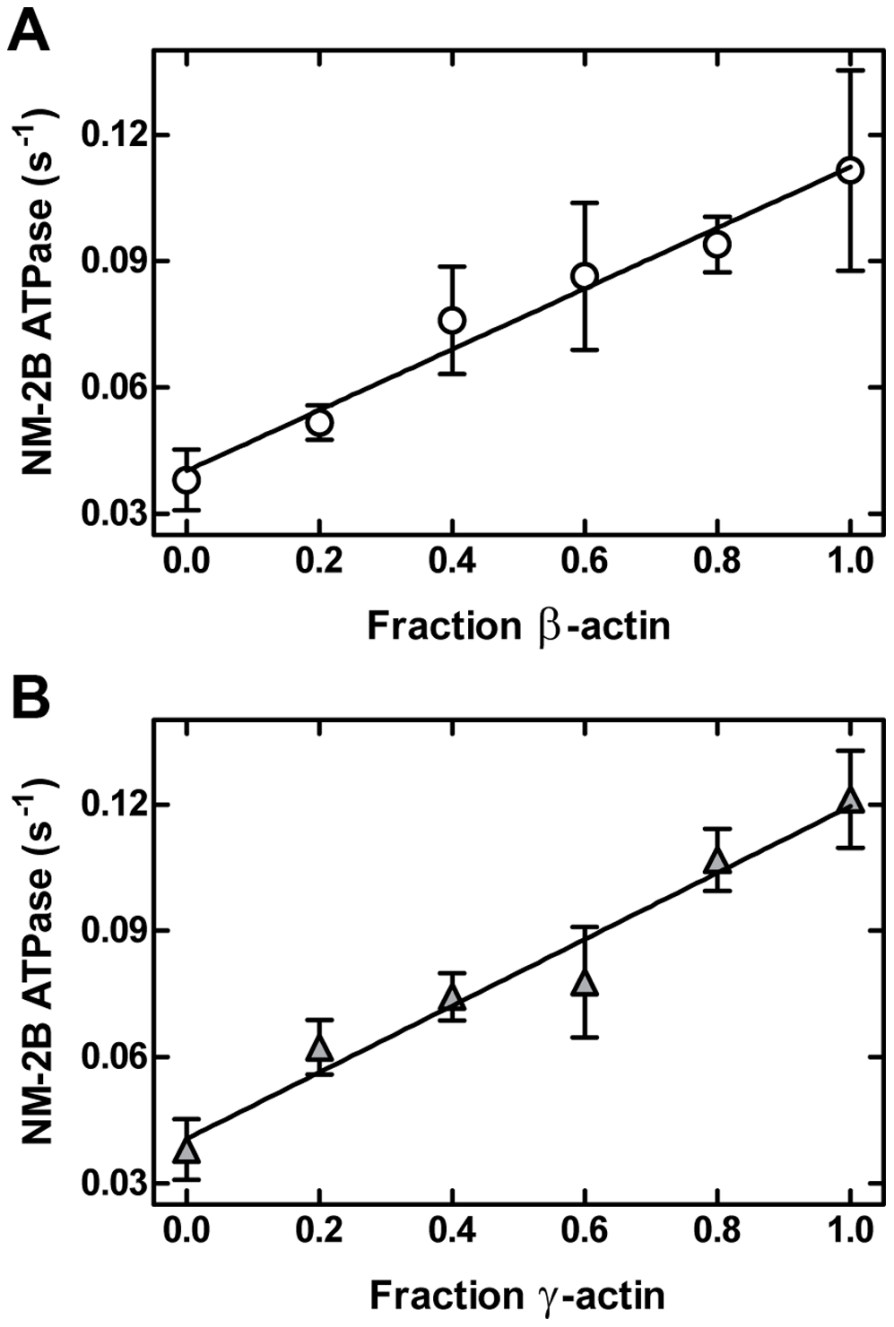
Activation of NM-2B ATPase rate by αβ- and αγ-actin copolymers. Copolymers with different mixing ratios were used to stimulate the ATPase activity of NM-2B. Measurements were performed using a final concentration of 20 µM F-actin.

To investigate the ability of isoactins to copolymerize, we performed fluorescence resonance energy transfer (FRET) assays using pairs of IAEDANS and IAF labeled isoactins. The isoactins copolymerized readily *in vitro*. Figure 6 shows the fluorescence spectrum of a mixture of the separately polymerized labeled actin isoforms, the spectrum obtained after copolymerization of IAEDANS-β-actin and IAF-γ-actin, and the associated difference spectrum. After copolymerization a marked reduction of the donor fluorescence and an increase of IAF fluorescence were observed. The filaments that result from mixing equimolar concentrations of labeled isoactins, display transfer efficiencies of 10–30% (Table 3). To analyze whether these hybrid filaments display an intermediate behavior or are dominated by the functional behavior of one isoactin, we examined how changes in isoform composition affect the activation of NM-2B ATPase activity. We copolymerized α-actin with β- and γ-actin respectively at increasing ratios (0, 0.2, 0.4, 0.6, 0.8, and 1.0) and used 20 µM copolymer solutions to stimulate NM-2B ATPase activity. Because the FRET copolymerization analysis did not show a preference for one particular isoactin to form homopolymers, we assume that the resulting filaments consist of randomly distributed α- and β-/γ-monomers. The catalytic activity which these mixed filaments support increases linearly with increasing β- or γ-actin content (Figure 7A and 7B).

**Table 3:** FRET efficiencies of copolymerized actin isoforms.

E (%)	IAEDANS-α-actin	IAEDANS-β-actin	IAEDANS-γ-actin
IAF-α-actin	29.5	33.7	23.1
IAF-β-actin	8.7	16.0	9.8
IAF-γ-actin	13.0	25.2	15.0

Transfer efficiencies (E) were measured as the relative donor fluorescence intensity in the presence (F_DA_) and absence (F_D_) of the acceptor E = (1−F_DA_/F_D_)/LD_A_ where LD_A_ is the labeling degree of the acceptor.

## Discussion

Our results show clear increases in actin-activation and functional competence for all nonsarcomeric myosins tested, when they were allowed to interact with cytoplasmic isoactins instead of α-actin. The preferred interaction of NM-2A, -2B and -2C1 with cytoplasmic β-actin and γ-actin fits well to the proteins' overlapping functions in cell migration, adhesion, cytokinesis, and cytoskeletal maintenance [3], [32], [57]. In regard to their interaction with β- or γ-actin, only NM-2A and NM-2B show no clear preference for either cytoplasmic isoactin. In our *in vitro* experiments, myosin-7A showed a distinct preference for γ-actin over β-actin. In their proper physiological context, myosin-7A, β- and γ-actin play a key role in the development, function and maintenance of cochlear hair cell stereocilia [14], [39]. Although the presence of both cytoplasmic actin isoforms is required for regular hair cell development, differences in the pattern of progressive hearing loss are observed upon their selective ablation. It was reported that γ-actin may be more abundant in particular hair cell structures and that the severity of progressive hearing loss can be related to the γ-actin concentration [14]. Further evidence for specific intracellular interactions between myosin-7A and γ-actin has been provided by studies of *MYO7A* gene defects [45], [46] and γ-actin mutations [21], [22], [23], [24], [25], [26], [27]. Our results pointing at a preferred interaction of the proteins provide an explanation why both sets of gene defects lead to similar phenotypes. NM-2C also plays a role in the function and maintenance of stereocilia. Mutations in NM-2C are associated with hereditary deafness (DFNA4) [37]. Genome-wide linkage analysis identified an autosomal-dominant mutation which causes a complex phenotype associated with peripheral neuropathy, myopathy, hoarseness, and hearing loss [58]. The results of our *in vitro* assays show that different from myosin-7A, NM-2C shows a clear preference for β-actin over γ-actin.

Although copolymerization of isoactins has only been studied for selected cell types, it is generally assumed that copolymers do not commonly exist *in vivo*. The basis for their discrete polymerization and localization remains to be determined, as FRET-based analysis confirms that *in vitro* isoactins do efficiently copolymerize [59]. Moreover, measurements with αβ-actin and αγ-actin copolymers indicate linear relationship between mixing ratio and isoform composition and the observed ATPase activity and the isoform composition of the copolymers. The behaviour of a hybrid F-actin can be reduced to the proportional representation of the isoforms suggesting that each actin acts independently from its neighbours.

Our results show that isoactins modulate the function of nonsarcomeric myosins in discrete ways. The observed differences in the interaction of individual actin and myosin isoforms are likely to be further enhanced by the impact of differential posttranslational modifications and interactions with regulatory actin binding proteins such as tropomyosin. Arginylation, which occurs in ∼40% of β-actin but not γ-actin, affects β-actin polymerization, localization, and lamella formation [60]. Furthermore, tropomyosin isoforms such as Tm5NM1 and TmBr3 show preferred interaction patterns with cytoplasmic isoactins and alter the recruitment and activity of NM-2 motors [61], [62].
